# Prevalence and associated factors of psychological distress among a national sample of in-school adolescents in Morocco

**DOI:** 10.1186/s12888-020-02888-3

**Published:** 2020-09-29

**Authors:** Supa Pengpid, Karl Peltzer

**Affiliations:** 1 ASEAN Institute for Health Development, Mahidol University, Salaya, Phutthamonthon, Nakhon Pathom, Thailand; 2 Department of Research Administration and Development, University of Limpopo, Polokwane, South Africa; 3 Department of Psychology, University of the Free State, Bloemfontein, South Africa

**Keywords:** Risk behaviour, Social factors, Psychological distress, Adolescents, Morocco

## Abstract

**Background:**

The goal of the study was to estimate the prevalence and correlates of psychological distress among adolescent school children in Morocco.

**Methods:**

Nationally representative cross-sectional data were analysed from 6745 adolescents (15 years median age) that responded to questions on a two-item measure of psychological distress from “2016 Morocco Global School-Based Student Health Survey (GSHS).”

**Results:**

The prevalence of psychological distress was 23.3, 18.0% among males and 29.2% among females. In adjusted logistic regression analysis, female sex, older age, bullying victimization, infrequently physically attacked, frequent participation in physical fights, having no close friends, frequent experience of hunger, parental emotional neglect, parental disrespect of privacy, school truancy, sedentary behaviour and having sustained a single or multiple serious injuries (past year) were associated with psychological distress. In addition, in unadjusted analysis, low peer support, parents never check homework, exposure to passive smoking, substance use (current tobacco use, current cannabis use and ever used amphetamine), frequent soft drink and frequent fast food consumption were positively and fruit and vegetable intake was negatively associated with psychological distress.

**Conclusion:**

Almost one in four students reported psychological distress and several associated factors were identified which can aid prevention and control strategies.

## Background

“Mental health conditions, including depression and anxiety, account for 16% of the global burden of disease and injury in people aged 10–19 years.” [1]. In children and adolescents, the “worldwide-pooled prevalence of mental disorders was 13.4%, including any anxiety disorder 6.5% and any depressive disorder 2.6%” [2]. “First onset of mental disorders usually occurs in childhood or adolescence” [3]. “Adolescence is a critical period characterised by vulnerability to psychological distress, and is therefore an important time for promotion of psychological well-being and early mental health intervention, in order to safeguard against the development of mental health issues” [4]. According to the American Psychological Association (APA) [5], psychological distress is “a set of painful mental and physical symptoms that are associated with normal fluctuations of mood in most people. It is thought to be what is assessed by many putative self-report measures of depression and anxiety.” For example, the Kessler Psychological Distress Scale includes “symptoms of depression, anxiety, stress, and somatic complaints” [6]. Psychological distress has been assessed with different scales, such as the “Psychological Distress Scale”, K-6 or K-10 [6, 7], the “General Health Questionnaire (GHQ-12)” [8] and several different psychological distress items, such as anxiety, loneliness, sadness and suicide plan [9], or no close friends, anxiety, loneliness, suicidal ideation and attempt [10].

The prevalence of psychological distress among adolescents in Eastern Mediterranean countries was 27.7% (≥2 items of no close friends, loneliness, anxiety, suicidal ideation, and suicide attempt) in Afghanistan [11], 16.8% (≥14 scores on GHQ-28) in Egypt [12], 17.7% (≥3 items of worthless, anxious, angriness, confusion, and insomnia) in Iran [13], and 17.2% probable depression and 21.4% probable anxiety (≥10 scores and ≥ 12 scores on the Hospital Anxiety and Depression Scale, respectively) in Pakistan [14]. The proportion of moderate to severe psychological distress (measured with the Kessler K-10) was 23.0% in a community-based study among adolescents (*N* = 1000) in Tanzania [15] and 10.5% among school-going adolescents in India [16], and the prevalence of psychological distress measured with the GHQ was 35.1% among adolescents in Ontario, Canada [17], and 24.2% among urban out of school adolescents (*N* = 480) in Nigeria [18]. In Zambia, the prevalence of psychological distress among adolescents was 15.7% (scoring 3 of 4 items, anxiety, loneliness, sadness, and suicide plan [9], in Tanzania among adolescents 20.6% had single psychological distress and 10.3% had multiple psychological distress (based on five items: anxiety, loneliness, no close friends, suicidal ideation and suicide attempt) [10], and in four Asian countries psychological distress was 32.9% (presence of any item: suicidal ideation, plan and suicide attempt, loneliness, sadness and anxiety) [19]. In a local survey among secondary school students in an urban area in Morocco (Tetouan), the most common mental problems “were memory problems, concentration difficulties, restlessness, fear, nervosity and feelings of inadequacy during interpersonal interactions.” [20]. There is a lack of national data on the prevalence and correlates of psychological distress among adolescents in Morocco. In order to prevent and control psychological distress in adolescent populations, it is vital to assess its prevalence and risk factors [21].

Factors associated with psychological distress among adolescents can be divided into social distresses, socio-environmental factors and health risk behaviours [22], and may include, as reviewed in Pengpid and Peltzer [10], sociodemographic factors (older age, female sex), social distresses (interpersonal violence), socio-environmental factors (experience of hunger, low peer and low parental support and school truancy) and health risk behaviours (substance use, sedentary behaviour, sexual behaviour and injury). This investigation aimed at estimating the prevalence and correlates of psychological distress among adolescents in Morocco.

## Methods

### Sources of data

Nationally representative cross-sectional data from the “2016 Morocco GSHS” were analyzed [23]. More detailed information on the survey methodology and the data can be accessed [23]; the overall response rate was 91% [23]. The sampling approach included a two stage sampling design, including schools and classes. All school students present in a selected class room were eligible to participate by filling in a self-administered anonymous questionnaire [23].

### Measures

The questionnaire (the questions, response options and coding) used is shown in supplementary file 1 [23].

### Outcome variable

*Psychological distress* was assessed with two items, 1) “During the past 12 months, how often have you been so worried about something that you could not sleep at night?” and 2) “During the past 12 months, how often have you felt lonely?” Response options included and were coded as “Never=0, Rarely=1, Sometimes=1, Most of the time=2, Always=3” Scores of the two items were summed and scores three or more were defined as psychological distress, following the scoring of other 2-item mental health screeners, the “Patient Health Questionnaire-2 (PHQ-2)” [24], and the “Generalized Anxiety Disorder-2 (GAD-2)” [25, 26]. Cronbach alpha for the 2-item “psychological distress” measure was 0.66 in this sample. The inter-item correlation was 0.44, and the item-total correlations ranged from .82 to .90.

### Co-variates

*Sociodemographic variables* included sex and age group.

*Psychosocial distress items* included the number of close friends, the number of days having been bullied in the past 30 days, the number of times having been physically attacked in the past 12 months, and the number of times having been involved in a physical fight in the past 12 months.

*Social-environmental variables* included, the frequency of feeling hungry in the past 30 days, the extend of peer support, parental disrespect of privacy, number of days exposed secondary smoke in the past 7 days, number of days truancy in the past 30 days, and parental emotional neglect was defined as never “parental or guardian understanding of your problems and worries? AND never parents or guardians really know what you were doing with your free time when you were not at school or work?” [27].

*Health risk behaviour items* included, current tobacco use, current cannabis use, ever used amphetamine, number of hours spent sedentary during leisure time, number of times seriously injured in the past 12 months, number of soft drinks consumed in a day, number of days having had fast food in the past week, and fruit and vegetable intake per day.

### Data analysis

Statistical analyses were done with “STATA software version 15.0 (Stata Corporation, College Station, Texas, USA),” taking into account the complex study design. The data were described using frequencies, means, and standard deviations. Pearson Chi-square tests were used for testing differences in proportions. In addition, Cronbach alpha coefficients were calculated and Principal Component Analysis with the 2-item psychological distress measure. Unadjusted and adjusted (with all co-variates) logistic regression analyses were used to assess predictors of psychological distress. Co-variates included age group, social distress (no close friends, being bullied, physically attacked, and participation in physical fight), social-environmental factors (experience of hunger, low peer support, lack of parental support, passive smoking, and school truancy), and health risk behaviours (tobacco use, cannabis use, amphetamine use, sedentary behaviour, injury, soft drink intake, fast food consumption, and fruit and vegetable intake). They were selected based on literature review [10]. Missing values (< 3.7% on any variable) were not included in the analysis.

## Results

### Factor analysis of the 2-item psychological distress measure

The Kaiser-Meyer-Olkin was .500, showing the adequacy of the sample, and the Bartlett’s Test of Sphericity (549,195.047, df = 1, *p* < 0.001) found that the factor analysis was justified. One component with eigenvalues larger than one (1.44) was extracted and named “psychological distress” explaining 72.21% of the variance (see Table 1).

**Table 1:** Factor loadings of the 2-item psychological distress measure among adolescents in Morocco

Psychological distress: 2 items	Component
	1
1. During the past 12 months, how often have you been so worried about something that you could not sleep at night?	.85
2. During the past 12 months, how often have you felt lonely?	.85
Eigenvalue	1.44
Percent of variance	72.21
Factor (items: 1 and 2): Psychological distress	

### Sample and psychological distress characteristics

The sample comprised 6745 school adolescents (15 years median age, 3 years interquartile range), 46.2% were female and 9.2% were mostly or always hungry. One in ten of the participants (10.2%) had no close friends, 19.8% were lonely, 18.2% had frequently been in a physical fight, 11.7% had frequently been attacked, and 14.7% had frequently been bullied. More than one in ten students (13.5%) reported current tobacco use, 6.3% current cannabis use, 7.8% had ever used amphetamine, 9.1% were daily exposed to secondary smoke, 17.2% had daily two or more soft drinks, 12.1% had multiple injuries (past year), 32.5% engaged in sedentary behaviour, 26.9% had on three or more days fast food in the past week and 33.0% had five or more servings of fruit and vegetables per day. One in ten of the students (10.1%) reported frequently truancy, 45.4% had low peer support, 24.1% experienced parental emotional neglect, 29.5% had parents who never checked on their home work, and 13.1% had parents who mostly or always disrespected their privacy. Almost one in four students (23.3%) reported psychological distress, 29.2% among females and 18.0% among males (see Table 2).

**Table 2:** Sample and psychological distress characteristics among adolescents in Morocco

Variable	Sample	Psychological distress		
		Both sexes	Male	Female
	N (%)	%	%	%
**Socio-demographics**				
**All**	6745	23.3	18.0	29.2
Age in years				
13 or less	1863 (26.8)	16.4	16.5	14.9
14–15	2212 (31.9)	21.9	14.4	28.6
16 or more	2558 (41.3)	30.3	21.2	41.0
**Psychosocial distress**				
No close friends	698 (10.2)	37.6	32.3	48.3
Bullied in past month				
0 days	3869 (61.5)	15.9	11.1	19.7
1 or 2 days	1511 (23.8)	29.3	20.0	40.5
3–30 days	955 (14.7)	45.5	36.7	59.6
Physically attacked in past year				
0 times	5080 (76.7)	20.2	14.1	25.6
time	795 (11.5)	32.5	26.6	40.1
2 or more times	793 (11.7)	38.8	31.6	53.1
In physical fight in past year				
0 times	64.2 (63.4)	21.7	14.1	26.7
1 time	18.4 (18.4)	23.2	15.1	35.2
2 or more times	17.4 (18.2)	31.8	27.4	46.5
**Social-environmental factors**				
Mostly/always feeling hungry	680 (9.2)	38.5	36.6	42.7
Low peer support	3085 (45.4)	25.6	18.9	34.0
Parental emotional neglect	1655 (24.1)	25.1	20.2	34.2
Parents never check home work	2029 (29.5)	25.7	20.2	33.5
Parents disrespect privacy	818 (13.1)	30.7	18.0	36.0
Passive smoking in past week				
0 days	3908 (59.0)	20.8	14.8	26.2
1–6 days	2104 (31.9)	24.7	18.4	32.2
All 7 days	580 (9.1)	38.2	30.7	43.4
School truancy (past month)				
0 days	4584 (69.3)	20.6	14.5	25.7
1–2 days	1317 (20.6)	28.4	20.0	37.5
3 or more days	644 (10.1)	36.1	31.6	48.7
**Health risk behaviours**				
Current tobacco use	913 (13.5)	31.9	29.8	40.4
Current cannabis use	453 (6.3)	34.1	37.1	37.6
Ever used amphetamine	464 (7.8)	37.2	36.6	47.9
Leisure time sedentary behaviour/day				
< 3 h	4574 (67.5)	21.2	16.3	24.9
3–4 h	1100 (18.7)	26.2	15.6	36.3
5–7 h	485 (8.4)	31.6	24.6	41.4
≥ 8 h	306 (5.4)	39.8	36.9	43.7
Injury in past 12 months				
0 times	4251 (68.4)	18.1	12.1	22.2
1 time	1206 (19.5)	29.7	23.2	39.0
2 or more times	735 (12.1)	39.5	32.2	58.6
Soft drink intake/day				
0	4488 (67.4)	23.5	16.6	29.4
1	1033 (15.3)	22.0	17.7	25.8
2	423 (6.6)	22.9	21.2	31.2
3 or more	706 (10.6)	27.1	23.5	31.3
Fast food consumption/week				
0 days	2486 (35.5)	22.3	15.9	26.7
1	1579 (23.7)	22.4	16.7	26.8
2	899 (13.8)	23.5	18.3	28.8
3–7 days	1718 (26.9)	27.0	21.5	35.6
Fruit and vegetable intake				
< 1 serving/day	560 (8.2)	32.3	24.2	43.0
1 or 2	2222 (34.1)	25.0	18.8	31.3
3 or 4	1591 (24.7)	20.5	14.1	26.7
5 or more	2158 (33.0)	22.0	17.9	25.5

### Associations with psychological distress among both sexes

Table 3 shows the unadjusted and adjusted associations between sociodemographic factors, social distress items, social-environmental factors and health risk behaviours with psychological distress. Compared to students aged 13 years or younger, older adolescents aged 16 years and older reported 129% (AOR = 2.29; 95% CI = 1.73–3.03) higher prevalence of psychological distress. Male adolescents were 70% (AOR = 0.30; 95% CI = 0.23–0.40) less likely having psychological distress than female adolescents do. Social distress factors associated with psychological distress were frequent bullying victimization (AOR = 2.92; 95% CI = 2.16–3.94), having no close friends (AOR = 1.98; 95% CI = 1.44–2.73), frequent involvement on physical fighting (AOR = 1.89; 95% CI = 1.34–2.67), and having been attacked once (AOR = 1.47; 95% CI = 1.08–2.00). Social-environmental factors associated with psychological distress were frequent truancy (AOR = 1.87; 95% CI = 1.23–2.83), frequent experience of hunger (AOR = 1.49; 95% CI = 1.07–2.08), parental emotional neglect (AOR = 1.37; 95% CI = 1.07–1.75), and parental disrespect of privacy (AOR = 1.33; 95% CI = 1.01–1.77). Regarding health risk behaviours, compared to students who had not sustained a serious injury in the past year, students who had multiple injuries were 122% (AOR = 2.22; 95% CI = 1.69–2.93) more likely to have psychological distress. Compared to students who were less than 3 h a day engaged in leisure-time sedentary behaviour, students who engaged eight or more hours a day in leisure-time sedentary behaviour had 71% (AOR = 1.71; 95% CI = 1.24–2.36) more likely psychological distress. In addition, in unadjusted analyses, low peer support, parents never check home work, exposure to passive smoking, substance use (current tobacco use, current cannabis use and ever used amphetamine), frequent soft drink and frequent fast food consumption were positively and fruit and vegetable intake was negatively associated with psychological distress (see Table 3).

**Table 3:** Associations with psychological distress in both sexes

Variable	Unadjusted Odds Ratio (95% CI)	*P*-value	Adjusted Odds Ratio (95% CI)	*P*-value
**Socio-demographics**				
Age in years				
13 or less	1 (Reference)		1 (Reference)	
14–15	1.46 (1.19, 1.79)	< 0.001	1.42 (1.07, 1.89)	0.016
16 or more	2.26 (1.89, 2.71)	< 0.001	2.29 (1.73, 3.03)	< 0.001
Gender				
Female	1 (Reference)		1 (Reference)	
Male	0.53 (0.45, 0.63)	< 0.001	0.30 (0.23, 0.40)	< 0.001
**Social distress**				
No close friends	2.53 (0.25, 3.24)	< 0.001	1.98 (1.44, 2.73)	< 0.001
Bullied in past month				
0 days	1 (Reference)		1 (Reference)	
1 or 2 days	2.22 (1.87, 2.62)	< 0.001	1.95 (1.58, 2.40)	< 0.001
3–30 days	4.70 (3.70, 5.98)	< 0.001	2.92 (2.16, 3.94)	< 0.001
Physically attacked in past year				
0 times	1 (Reference)		1 (Reference)	
1 time	1.89 (1.51, 2.37)	< 0.001	1.47 (1.08, 2.00)	0.015
2 or more times	2.63 (2.13, 3.23)	< 0.001	1.02 (0.72, 1.46)	0.987
In physical fight in past year				
0 times	1 (Reference)		1 (Reference)	
1 time	1.00 (0.83, 1.21)	0.989	1.20 (0.89, 1.60)	0.232
2 or more times	1.66 (1.34, 2.04)	< 0.001	1.89 (1.34, 2.67)	< 0.001
**Social-environmental factors**				
Mostly/always feeling hungry	2.30 (1.78, 2.98)	< 0.001	1.49 (1.07, 2.08)	0.019
Low peer support	1.27 (1.11, 1.45)	< 0.001	1.15 (0.97, 1.35)	0.098
Parental emotional neglect	1.29 (1.09, 1.52)	0.003	1.37 (1.07, 1.75)	0.014
Parents never check home work	1.27 (1.02, 1.58)	0.031	1.11 (0.90, 1.36)	0.326
Parents disrespect privacy	1.27 (1.07, 1.50)	0.006	1.33 (1.01, 1.77)	0.045
Passive smoking in past week				
0 days	1 (Reference)		1 (Reference)	
1–6 days	1.18 (1.04, 1.33)	0.010	0.89 (0.67, 1.17)	0.388
All 7 days	2.17 (1.78, 2.64)	< 0.001	1.19 (0.82, 1.71)	0.362
School truancy (past month)				
0 days	1 (Reference)		1 (Reference)	
1–2 days	1.49 (1.28, 1.74)	< 0.001	1.37 (1.08, 1.74)	0.009
3 or more days	2.35 (1.81, 3.04)	< 0.001	1.87 (1.23, 2.83)	0.004
**Health risk behaviours**				
Current tobacco use	1.67 (1.34, 2.07)	< 0.001	0.68 (0.48, 1.01)	0.098
Current cannabis use	2.07 (1.56, 2.74)	< 0.001	0.96 (0.57, 1.61)	0.877
Ever used amphetamine	2.34 (1.86, 2.94)	< 0.001	1.58 (0.91, 2.75)	0.100
Leisure time sedentary behaviour/day				
< 3 h	1 (Reference)		1 (Reference)	
3–4 h	1.29 (1.09, 1.53)	0.003	1.04 (0.73, 1.48)	0.820
5–7 h	1.74 (1.42, 2.14)	< 0.001	1.68 (1.23, 2.30)	< 0.001
≥ 8 h	2.70 (2.10, 3.46)	< 0.001	1.71 (1.24, 2.36)	< 0.001
Injury in past 12 months				
0 times	1 (Reference)		1 (Reference)	
1 time	2.00 (1.74, 2.29)	< 0.001	1.50 (1.20, 1.87)	< 0.001
2 or more times	3.23 (2.73, 3.82)	< 0.001	2.22 (1.69, 2.93)	< 0.001
Soft drink intake/day				
0	1 (Reference)		1 (Reference)	
1	0.95 (0.74, 1.22)	0692	0.85 (0.61, 1.19)	0.335
2	1.21 (0.91, 1.63)	0.194	0.94 (0.65, 1.36)	0.756
3 or more	1.28 (1.06, 1.54)	0.011	1.00 (0.60, 1.64)	0.986
Fast food consumption/week				
0 days	1 (Reference)		1 (Reference)	
1	0.96 (0.80, 1.14)	0.618	0.92 (0.67, 1.26)	0.600
2	1.04 (0.90, 1.20)	0.593	0.86, 0.65, 1.13)	0.282
3–7 days	1.52 (1.19, 1.94)	< 0.001	1.04 (0.75, 1.44)	0.828
Fruit and vegetable intake				
< 1 serving/day	1 (Reference)		1 (Reference)	
1 or 2	0.66 (0.51, 0.86)	0.003	0.99 (0.74, 1.33)	0.965
3 or 4	0.51 (0.39, 0.67)	< 0.001	0.86 (0.61, 1.20)	0.374
5 or more	0.55 (0.42, 0.72)	< 0.001	0.88 (0.61, 1.28)	0.499

*CI* Confidence Interval

### Associations with psychological distress among boys and girls

Compared to 13 or less year-old girls, 16 or more year-old girls were 3.5 times more likely to have psychological distress. Among both boys and girls, frequent bullying victimization, frequently having been in a physical fight, having no close friends, frequent truancy and frequent injury increased the odds for psychological distress. Boys who frequently experienced hunger were 114% (AOR = 2.14; 95% CI = 1.31–3.48) more likely to have psychological distress, and girls who had low peer support were 23% (AOR = 1.23; 95% CI = 1.05–1.44) and engaged 8 or more hours in leisure-time sedentary behaviour were 91% (AOR = 1.91; 95% CI = 1.14–3.20) more likely to have psychological distress (see Table 4).

**Table 4:** Associations with psychological distress among males and females

Variable	Male		Female	
	Adjusted Odds Ratio (95% CI)	*p*-value	Adjusted Odds Ratio (95% CI)	*p*-value
**Socio-demographics**				
Age in years				
13 or less	1 (Reference)		1 (Reference)	
14–15	0.77 (0.46, 1.29)	0.321	2.12 (1.48, 3.03)	< 0.001
16 or more	1.23 (0.75, 2.02)	0.414	3.52 (2.50, 4.95)	< 0.001
**Social distress**				
No close friends	2.88 (1.73, 4.81)	< 0.001	1.61 (1.14, 2.28)	0.007
Bullied in past month				
0 days	1 (Reference)		1 (Reference)	
1 or 2 days	1.61 (1.08, 2.39)	0.020	2.24 (1.60, 3.15)	< 0.001
3–30 days	2.50 (1.63, 3.82)	< 0.001	3.41 (2.24, 5.18)	< 0.001
Physically attacked in past year				
0 times	1 (Reference)		1 (Reference)	
1 time	1.64 (1.00, 2.70)	0.051	1.17 (0.73, 1.88)	0.516
2 or more times	1.02 (0.67, 1.57)	0.921	1.19 (0.62, 2.31)	0.594
In physical fight in past year				
0 times	1 (Reference)		1 (Reference)	
1 time	1.12 (0.77, 1.64)	0.549	1.38 (0.94, 2.03)	0.099
2 or more times	1.98 (1.33, 2.96)	< 0.001	1.86 (1.21, 2.87)	0.005
**Social-environmental factors**				
Mostly/always feeling hungry	2.14 (1.31, 3.48)	0.002	1.05 (0.69, 1.61)	0.805
Low peer support	0.99 (0.77, 1.27)	0.915	1.23 (1.05, 1.44)	0.013
Parental emotional neglect	1.28 (0.91, 1.81)	0.404	1.43 (0.98, 2.08)	0.063
Parents never check home work	1.36 (0.99, 1.86)	0.057	0.92 (0.68, 1.26)	0.610
Parents disrespect privacy	1.20 (0.78, 1.85)	0.404	1.32 (0.88, 2.00)	0.182
Passive smoking in past week				
0 days	1 (Reference)		1 (Reference)	
1–6 days	0.88 (0.67, 1.14)	0.329	0.90 (0.65, 1.25)	0.519
All 7 days	1.15 (0.77, 1.71)	0.494	1.27 (0.76, 2.12)	0.353
School truancy (past month)				
0 days	1 (Reference)		1 (Reference)	
1–2 days	1.58 (1.15, 2.17)	0.006	1.28 (0.92, 1.79)	0.146
3 or more days	2.12 (1.28, 3.51)	0.004	2.01 (1.04, 3.89)	0.039
**Health risk behaviours**				
Current tobacco use	0.81 (0.53, 1.24)	0.326	0.50 (0.19, 1.28)	0.147
Current cannabis use	0.94 (0.55, 1.58)	0.804	1.09 (0.30, 3.90)	0.896
Ever used amphetamine	1.36 (0.75, 2.47)	0.303	1.96 (0.57, 6.76)	0.285
Leisure time sedentary behaviour/day				
< 3 h	1 (Reference)		1 (Reference)	
3–4 h	0.75 (0.47, 1.19)	0.214	1.26 (0.83, 1.90)	0.273
5–7 h	1.28 (0.88, 1.87)	0.193	2.06 (1.25, 3.41)	0.005
≥ 8 h	1.36 (0.80, 2.31)	0.250	1.91 (1.14, 3.20)	0.014
Injury in past 12 months				
0 times	1 (Reference)		1 (Reference)	
1 time	1.55 (1.18, 2.04)	0.002	1.37 (0.95, 1.97)	0.090
2 or more times	1.75 (1.27, 2.42)	< 0.001	3.09 (1.73, 5.48)	< 0.001
Soft drink intake/day				
0	1 (Reference)		1 (Reference)	
1	1.18 (0.76, 1.84)	0.459	0.70 (0.50, 0.99)	0.043
2	1.56 (1.00, 2.45)	0.050	0.63 (0.33, 1.20)	0.160
3 or more	1.13 (0.56, 2.29)	0.729	0.89 (0.52, 1.52)	0.664
Fast food consumption/week				
0 days	1 (Reference)		1 (Reference)	
1	1.08 (0.72, 1.65)	0.689	0.80 (0.52, 1.22)	0.293
2	0.98 (0.67, 1.43)	0.905	0.77 (0.47, 1.27)	0.302
3–7 days	0.89 (0.62, 1.27)	0.515	1.13 (0.75, 1.71)	0.549
Fruit and vegetable intake				
< 1 serving/day	1 (Reference)		1 (Reference)	
1 or 2	1.11 (0.65, 1.91)	0.705	0.99 (0.62, 1.59)	0.573
3 or 4	0.95 (0.58, 1.57)	0.852	0.86 (0.56, 1.32)	0.494
5 or more	1.16 (0.67, 2.00)	0.595	0.79 (0.51, 1.23)	0.294

*CI* Confidence Interval

## Discussion

The current study aimed at estimating the prevalence and correlates of psychological distress in school adolescents in Morocco. The prevalence of past 12-month psychological distress (23.3%) in this study, which is similar to some previous studies among adolescents, e.g., in Tanzania (23.0%) [15], Nigeria (24.2%) [18], lower than in Afghanistan (27.7%) [11], in United Arab Emirates (28% anxiety disorders) [28], in Lebanon (Beirut) (26.1% mental disorders [29], in Canada (35.1%) [17], and higher than in Egypt (16.8%) [12], India (10.5%) [16], and Iran (17.7%) [13]. Other previous studies among adolescents in the Morocco seem to confirm that psychological distress is common [20], calling for strategies and programmes to prevent and control psychological distress in this adolescent population in Morocco.

The study showed that being female increased the odds for psychological distress, which was also found in some previous investigations [11]. Generally, “girls are more likely than boys to report internalising problems such as psychological distress, depression, and anxiety.” [4, 30, 31]. The study showed that older age, in particular among girls, increased the odds for psychological distress. Similar results were found in a study among adolescents in India [16]. Possible reasons for higher psychological distress among older than younger adolescents include increasing demands, physical and psychosocial changes [4, 32, 33]. In addition, older adolescents in this study were more likely to be injured (*p* = 0.009), engage in substance use (tobacco use: *p* < 0.001, cannabis use: *p* = 0.008, amphetamine use: *p* = 0.003), truancy (*p* < 0.001), sedentary behaviour (*p* < 0.001), passive smoking (*p* < 0.001), had no close friends (*p* < 0.001), frequent fast food intake (*p* = 0.003) and ate less fruit and vegetables (*p* < 0.001) than younger adolescents, which may have contributed to increased psychological distress among older adolescents.

In line with former research findings [9, 10, 19, 34], this survey showed that having social distress, such as having no close friends, bullying victimization, infrequently physically attacked and frequently involved in physical fighting increased the odds for psychological distress. Students exposed to interpersonal violence victimization may worry about further or future victimization increasing psychological distress. In addition, our study findings demonstrate that students who had been frequently bullied had the highest odds for psychological distress, which concurs with former research [35]. This finding may highlight the relevance of anti-bullying programme activities in order to ameliorate psychological distress. “The design and implementation of school-based anti-bullying programmes in the Arab world to reduce the harmful effects of bullying are lacking.” [36] However, Morocco has “implemented coordinated national action plans to address violence against children, enforced legislation to protect victims, and promoted programmes aimed at changing societal beliefs and attitudes around violence.” [37]

Several social-environmental factors (experience of hunger, in particular among boys, parental emotional neglect, parental disrespect of privacy, infrequent truancy and in unadjusted analysis passive smoking, low peer support, in particular among girls, and parents never check homework) were found associated with psychological distress. These results are consistent with various previous investigations [9, 10, 19, 38, 39] and call for programmes improving parental and peer support and food security. A previous review provides evidence that “parental training and school-based interventions can reduce symptoms of common mental disorders in adolescents” [40].

In terms of health risk behaviours, high sedentary behaviour (in particular among girls) and having experienced single and multiple serious injuries, and in unadjusted analysis frequent fast food consumption, infrequent fruit and vegetable intake, frequent soft drink, having no close friends, and substance use increased the odds for having psychological distress. These findings concur with previous studies [10, 17, 41–45]. Since this study did not assess the type of sedentary behaviour, for example social media use, we are not able to show the potentially negative effects of social media use on psychological distress [46]. In a systematic review among adolescents [47] found that sedentary behaviour was associated with poor mental health and psychological distress, which may be explained by “the beneficial pathophysiological, social and general health effects of being active may be omitted when sedentary, which may have a negative impact on mental health.” Another possible mechanism by which sedentary behaviour may increase psychological distress is via inflammatory processes [48]. For example, in a randomized controlled intervention, “a one-week sedentary behaviour-inducing intervention had deleterious effects on anxiety in an active, young adult population” [49]. The association between injury occurrence in the past 12 months and psychological distress in the past 12 months may be explained by “the injury occurrence being considered a particularly impactful stressful life event, and experiences of stressful life events have been strongly associated with prospective anxiety symptom development” [50].

### Study limitations

Study limitations include that this investigation was limited because of its cross-sectional design, the inclusion of only school adolescents as well as the self-report of the data. An additional limitation was that the GSHS in Morocco only assessed psychological distress with two items which may not reflect a standardized scale nor a diagnostic interview, and did not assess help seeking behaviours for psychological distress.

## Conclusion

The study found among school-going nationally representative adolescents in Morocco that almost one in four students reported psychological distress. Several correlates for psychological distress were identified, including female sex, having no close friends, older age, bullying victimization, infrequently physically attacked, frequent participation in physical fights, frequent experience of hunger, parental emotional neglect, parental disrespect of privacy, school truancy, sedentary behaviour and having sustained a single or multiple serious injuries (past year), which can potentially guide interventions to prevent psychological distress in this adolescent school population.

## Supplementary information

**Additional file 1.** Variable description.

## Data Availability

The data for the current study are publicly available at the World Health Organization NCD Microdata Repository (URL: https://extranet.who.int/ncdsmicrodata/index.php/catalog).

## Abbreviations

GSHS: Global School-Based Student Health Survey
STATA: Statistics and data
