# Supplementary material for: Prevalence and associated factors of psychological distress among a national sample of in-school adolescents in Morocco
Source: BMC Psychiatry. 2020 Sep 29;20:475. doi: 10.1186/s12888-020-02888-3 (PMC7526246; doi:10.1186/s12888-020-02888-3)
Supplement: Supplementary file 1 — Additional file 1. Variable description. [file 12888_2020_2888_MOESM1_ESM.docx]

Supplementary file 1: Variable description

| **Variables** | **Question** | **Response options (coding scheme)** |
| --- | --- | --- |
| Anxiety | “During the past 12 months, how often have you been so worried about something that you could not sleep at night?” | “1=never to 5=always” |
| Loneliness | “During the past 12 months, how often have you felt lonely?” | “1=never to 5=always” |
| Age | “How old are you?” | “11 years old or younger to 18 years old or older” |
| Sex | “What is your sex?” | “Male, Female” |
|  | *Social distress* |  |
| No close friends | “How many close friends do you have?” | “1 = 0 to 4 = 3 or more (coded 1+=0, 0=1)” |
| Bullied | “During the past 30 days, on how many days were you bullied?” | “1=0 days to 7=All 30 days” |
| Physically attacked | “During the past 12 months, how many times were you physically attacked?” | “1=0 times to 8=12 or more times” |
| Physical fights | “During the past 12 months, how many times were you in a physical fight?” | “1=0 times to 8=12 or more times” |
|  | *Social-environmental factos* |  |
| Hunger | “During the past 30 days, how often did you go hungry because there was not enough food in your home?" | “1 = never to 5 = always (coded 1-3=0 and 4-5=1)” |
| Peer support | “During the past 30 days, how often were most of the students in your school kind and helpful?” | “1=never to 5=always (coded 1-2=1, 3–5=0)” |
| Parental supervision | “During the past 30 days, how often did your parents or guardians check to see if your homework was done?” | “1=never to 5=always (coded 1=1 and 2–5=0)” |
| Parental emotional neglect | “During the past 30 days, how often did your parents or guardians understand your problems and worries?” | “1=never to 5=always (coded 1=1 and 2-5=0)” |
|  | “During the past 30 days, how often did your parents or guardians really know what you were doing with your free time?” | “1=never to 5=always (coded 1-2=1 and 3-5=0)” |
| Parental disrespect for privacy | “During the past 30 days, how often did your parents or guardians go through your things without your approval?” | “1=never to 5=always (coded 1–3=0 and 4–5=1)” |
| Passive smoking | “During the past 7 days, on how many days have people smoked in your presence?” | “1=0 days to 5=all 7 days” |
| School truancy | “During the past 30 days, on how many days did you miss classes or school without permission?” | “1=0 days to 5= 10 or more days” |
|  | *Health risk behaviours* |  |
| Current tobacco use | “During the past 30 days, on how many days did you smoke cigarettes/use any tobacco products other than cigarettes, such as Shisha, Tabac à snifer, Kala?” | “1=0 days to 7=All 30 days (coded 1=0 and 2-7=1)” |
| Current cannabis use | “During the past 30 days, how many times have you used marijuana?” | “1=0 times to 5=20 or more times (coded 1=0 and 2-5=1)” |
| Amphetamine use | “During your life, how many times have you used amphetamines or methamphetamines?” | “1=0 times to 5=20 or more times (coded 1=0 and 2-5=1)” |
| Leisure-time sedentary behavior | “How much time do you spend during a typical or usual day sitting and watching television, playing computer games, talking with friends, or doing other sitting activities, such as using the computer or cell phone?” | “1=Less than 1 hour per day… 3= 3 to 4 hours per day …6=8 or more hours a day” |
| Soft drink intake | “During the past 30 days, how many times per day did you usually drink carbonated soft drinks, such as Coca-Cola or Fanta? (Do not include diet soft drinks.)” | “1=0 times to 7=5 or more times (coded 1-2=0 and 3-7, 2 or more times)” |
| Fruits | “During the past 30 days, how many times per day did you usually eat fruit such asapples, oranges, and bananas?” | “1=I did not eat fruit during the past 30 days to 7=5 or more times per day” |
| Vegetables | “During the past 30 days, how many times per day did you usually eat vegetables, such as potatoes or carrots?” | “I did not eat vegetables during the past 30 days to 7=5 or more times per day ” |
| Injury | “During the past 12 months, how many times were you seriously injured?” | “1=0 times to 8=12 or more times (coded 1=0 and 2–8=1)” |
